# Deterministic Factors Overwhelm Stochastic Environmental Fluctuations as Drivers of Jellyfish Outbreaks

**DOI:** 10.1371/journal.pone.0141060

**Published:** 2015-10-20

**Authors:** Lisandro Benedetti-Cecchi, Antonio Canepa, Veronica Fuentes, Laura Tamburello, Jennifer E. Purcell, Stefano Piraino, Jason Roberts, Ferdinando Boero, Patrick Halpin

**Affiliations:** 1 Department of Biology, University of Pisa, CoNISMa, Via Derna 1, Pisa, Italy; 2 Institut de Ciències del Mar, Consejo Superior de Investigaciones Científicas, ICM-CSIC, Passeig Marítim de la Barceloneta, 37–49, 08003 Barcelona, Spain; 3 Università del Salento, CoNISMa via Monteroni, 73100 Lecce, LE, Italy; 4 Western Washington University, Shannon Point Marine Center, Anacortes, Washington 98221, United States of America; 5 Marine Geospatial Ecology Laboratory, Nicholas School of the Environment, Duke University, Durham, North Carolina, United States of America; 6 CNR-ISMAR, Genova, Italy; GEOMAR: Helmholtz Center for Ocean Research, GERMANY

## Abstract

Jellyfish outbreaks are increasingly viewed as a deterministic response to escalating levels of environmental degradation and climate extremes. However, a comprehensive understanding of the influence of deterministic drivers and stochastic environmental variations favouring population renewal processes has remained elusive. This study quantifies the deterministic and stochastic components of environmental change that lead to outbreaks of the jellyfish *Pelagia noctiluca* in the Mediterranen Sea. Using data of jellyfish abundance collected at 241 sites along the Catalan coast from 2007 to 2010 we: (1) tested hypotheses about the influence of time-varying and spatial predictors of jellyfish outbreaks; (2) evaluated the relative importance of stochastic vs. deterministic forcing of outbreaks through the environmental bootstrap method; and (3) quantified return times of extreme events. Outbreaks were common in May and June and less likely in other summer months, which resulted in a negative relationship between outbreaks and SST. Cross- and along-shore advection by geostrophic flow were important concentrating forces of jellyfish, but most outbreaks occurred in the proximity of two canyons in the northern part of the study area. This result supported the recent hypothesis that canyons can funnel *P*. *noctiluca* blooms towards shore during upwelling. This can be a general, yet unappreciated mechanism leading to outbreaks of holoplanktonic jellyfish species. The environmental bootstrap indicated that stochastic environmental fluctuations have negligible effects on return times of outbreaks. Our analysis emphasized the importance of deterministic processes leading to jellyfish outbreaks compared to the stochastic component of environmental variation. A better understanding of how environmental drivers affect demographic and population processes in jellyfish species will increase the ability to anticipate jellyfish outbreaks in the future.

## Introduction

Extreme events such as droughts, storms and floods are becoming more frequent with climate change. These environmental changes may provoke extreme ecological responses in populations and species assemblages that may result in severe impacts to natural ecosystems [1–4]. Population outbreaks are examples of extreme ecological events that may be driven by extreme climate conditions [5, 6]. Outbreaks occur when the alignment in space and time of certain environmental drivers (abiotic and biotic), result in particularly favourable conditions for population renewal processes [7]. Defining these conditions quantitatively and understanding when and where they will occur are key tasks to forecast population outbreaks. This endeavour is particularly challenging, because episodic events may be inherently unpredictable [8, 9].

The environmental envelope that provides favourable conditions for population renewal is the result of a combination of deterministic and chance events [7]. Deterministic events reflect what is known about population responses to environmental drivers. The relationship between temperature and organismal growth and development is an example [10]. Thus, global warming provides a deterministic vector of environmental change. Unfortunately, for many other drivers our understanding of how they impinge on natural populations is limited, let alone our understanding of the compounded effects of multiple drivers [11, 12]. Chance events reflect our unknowns, which can be treated as stochastic vectors of environmental change.

Deterministic events can be identified by relating environmental drivers to species outbreaks through appropriate statistical techniques, such as multiple regression and related approaches. Although the evidence remains correlative, testing mechanistic hypotheses increases inferential strength [13, 14]. Addressing stochastic events requires randomization procedures, so that observed probabilities of occurrence of outbreaks can be compared to those expected from chance alone. The environmental bootstrap method has been specifically designed for this purpose [7]. The method consists of bootstrapping relatively short (5–10 yrs) time series of environmental variables to determine the probability that a particular set of conditions (e.g. the combination of high nutrients and extreme temperatures that can favour outbreaks of exotic species) [15] will occur by chance alone. In its original formulation the procedure used mechanistic response functions to translate environmental extremes into meaningful biological responses. In principle, the method is not restricted to mechanistic response functions, but can be extended to any class of models that relate biological variables to environmental data. For example, one might use a multiple regression approach to determine the circumstances under which species outbreaks become more likely and couple this statistical model with the environmental bootstrap method to determine the probability that the event will occur by chance alone, at any place and time for which a short history of environmental data is available.

Jellyfish blooms are examples of species outbreaks that may have adverse effects on fisheries, human health and tourism, with associated social costs and are therefore of great concern to scientists, policy makers and the public at large [16–19]. Although the drivers of increasingly frequent jellyfish blooms are not fully understood, they likely involve a combination of global climate events and local anthropogenic stressors. Climate events include global warming, which may positively affect jellyfish population processes and vital rates [20, 21]. Recent studies have related jellyfish oscillations with ocean-atmospheric variability, including lunar cycles [22]. Local drivers that may cause jellyfish blooms include (but are not limited to) changes in food web structure owing to the depletion of potential predators and competitors from overfishing, human modification of coastal habitats, including the proliferation of artificial structures that may provide habitats for jellyfish benthic stages, shipping and eutrophication [16, 23, 24].

This study quantifies the deterministic and stochastic components of environmental change that lead to favourable conditions for jellyfish outbreaks, recorded as mass strandings along the Catalan coast in Spain. We focus on *Pelagia noctiluca* (Forsskål), a common holoplanktonic scyphozoan with a wide geographic distribution that extends from the warm and temperate waters of the world’s oceans up to the North Sea [25, 26]. Blooms of *P*. *noctiluca* have been recorded for over two centuries in the Mediterranean with a periodicity of approximately 12 years, apparently in response to the occurrence of warm winters [27]. This positive association with temperature explains the observed trend of increasing frequency of outbreaks of *P*. *noctiluca* in the Mediterranean [28, 29].

We employ a Bayesian analysis to relate spatio-temporal dynamics of *P*. *noctiluca* blooms to a set of explanatory environmental variables, coupling the resulting model with the environmental bootstrap method to assess the relative importance of deterministic vs. stochastic chance events leading to jellyfish outbreaks. We use sea surface temperature (SST), primary production (PP), chlorophyll-*a* (chlorophyll), geostrophic current velocities and distance from the nearest marine canyon as environmental predictors of jellyfish outbreaks. SST provides a surrogate measure for environmental energy, which is important for many metabolic functions [30, 31]. PP reflects the rate of carbon fixation by the autotrophic community and is an indicator of food availability for gelatinous zooplankton [32]. Chlorophyll is the net biomass of primary producers after removal processes such as grazing have been accounted for, and may also provide an indication of food availability for jellyfish [33]. Zonal and meridional current velocities are included as potentially important predictors of jellyfish advection. We hypothesized a positive relation between these drivers and jellyfish outbreaks. Furthermore, we included distance from the nearest canyon as a covariate to test the recent hypothesis that when occurring near the coast, these physiographic features of the deep sea environment can bring *P*. *noctiluca* near shore during upwelling from mesopelagic source populations [34]. The specific objectives of this study were to: (1) test for the significance of hypothesized relationships between environmental predictors and jellyfish outbreaks, (2) evaluate the relative importance of stochastic vs. deterministic forcing of outbreaks, and (3) quantify the return times of extreme jellyfish outbreaks along the Catalan coast.

## Materials and Methods

### Data

We use a dataset consisting of semi-quantitative measures of the abundance of *P*. *noctiluca* jellyfish at 241 sites along the Catalan coast (Spain). Access to field sites was provided by the Catalan Water Agency and the Catalan Autonomic Administration. The data were collected daily through a Citizen Science program under the supervision of the Catalan Water Agency from May to September from 2007 to 2010 [34]. We defined an outbreak as the occurrence of at least one stranded jellyfish m^-2^. Species outbreaks, especially in gelatinous plankton, imply sudden appearances and virtual absences for prolonged periods [18]. Hence, the dataset based on daily occurrence of jellyfish blooms had many zeros, precluding a meaningful spatio-temporal analysis of daily outbreaks. To mitigate this problem we computed the number of days with outbreaks over monthly periods for each year at each site. This resulted in 20 data points (four years x five months) for each of the 241 sites. Although jellyfish blooms that are observed over short time intervals (days or weeks) may be part of the same outbreak, for simplicity, we refer to the number of days in a month in which an outbreak was observed as the number of outbreaks.

Quantifying jellyfish outbreaks from stranding data may be problematic if counts are not temporally independent. In our case sampling sites were cleaned daily when more than 10 jellyfish were stranded per beach. Therefore, our definition of an outbreak involving one or more jellyfish m^-2^ ensured that the daily measures reflected new animals, effectively preventing the accumulation of counts through subsequent days. Furthermore, stranding data are not necessarily representative of jellyfish densities in the nearshore environment. For example, small animals and ephyrae would be undetected in beach surveys. A comparison of the incidence of outbreaks based on stranding data with counts made from the boat for a subset of sites and sampling dates indicated that stranding data generally reflected abundance patterns in the nearshore environment (S1 Fig).

Daily values of SST, PP, chlorophyll and geostrophic current velocities were downloaded using the Marine Geospatial Ecology Tools (Roberts et al. 2010), from publically available databases from 1 May to 30 September for the years 2004–2010 (S1 Table and S1 References). These data matched the seasons of jellyfish sampling, but also extended earlier in time to enable the characterization of the recent environmental history of each site, which was necessary to implement the environmental bootstrap analysis. When data were not available for a site, the average value from nearby cells was used. Distance from the nearest canyon was determined for each site using the marmap library in R [35].

### Bayesian hierarchical analysis

We used a Bayesian hierarchical analysis based on the INLA approach (Integrated Nested Laplace Approximation) to model the number of outbreaks [36]. Spatial dependencies were modelled through a Gaussian Markov Random Field (GMRF) and temporal autocorrelation as an autoregressive AR(1) process. We implemented the GMRF representation using the SPDE method (Stochastic Partial Differential Equation) [37, 38].

Briefly, a GMRF is a spatial process that models the spatial dependence of data through the definition of a neighbourhood structure based on geographic coordinates, so that only sites within a certain distance from each other are spatially correlated, whereas sites further distances apart are spatially independent. The SPDE approach represents the GMRF through a fine mesh triangulation (e.g., using Delaunay triangulation) of the study area where the latent variable (the true unobserved value of the response variable) is modelled at the vertices of the triangles. Sampling locations that fall within a triangle (i.e. that are not on a vertex) take the average value of the three nearby vertices. We specified a mesh with 741 vertices with the greatest density of triangles located along the coast corresponding to the sampling sites (S2 Fig). The outer triangles were uninformative and had lower resolution. Obviously, triangles falling on land were meaningless.

Spatial dependencies were modelled through the Matérn spatial covariance function with scale and smoothness parameters, respectively *κ* and *ν*, which define the empirically derived relationship $\rho =\sqrt{8\nu /\kappa }$, where *ρ* is the distance at which spatial correlation becomes close to 0.1, for each *ν* (with *ν* = 1 in calculations).

In the spatio-temporal context, the GMRF **ζ**_*t*_ *=* (*ζ*(*s*_1_,*t*),…,*ζ*(*s*_*n*_,*t*)), with *s*_1_ to *s*_*n*_ sampling sites, is modelled as a first order autoregressive process:

$$\zeta_{t}=a\zeta_{t-1}+\omega_{t}$$ (1a)

$$\omega_{t}\sim N\left(0,Q^{-1}\right),$$ (1b)

where **Q**_*s*_ is a sparse precision matrix of size *n*, corresponding to the number of vertices of the domain triangulation and with $\zeta_{1}\sim N\left(0,Q_{s}^{-1}/(1-a^{2})\right)$. **Q**_*s*_ = Σ^−1^, where $\Sigma =\sigma_{w}^{2}C(h)$ is the covariance matrix with *C(h)* being the Matérn spatial correlation function for the Euclidean distance lags *h*. This formulation assumes that **Q**_*s*_ does not change through time, which means that the GMRF has a constant correlation structure and changes through time according to a first order autoregressive process.

The number of outbreaks at time *t* (with time defined by a specific year and month combination) at the *i-*th site, was modelled as a Poisson process *y*_*it*_ ~ Poisson (*λ*_*it*_), with mean *λ*_*it*_ and with the linear predictor defined on the logarithmic scale as:

$$\eta_{it}=\text{log}(\lambda_{it})=z_{it}\beta +\sum_{j=1}^{G}B_{ij}\zeta_{t}$$ (2)

where **z**_*it*_ is a vector of *p* covariates for site *i* at time *t*, ***β*** is the vector of *p* coefficient parameters (including the intercept) and **B**_*ij*_ is the sparse matrix that maps the GMRF **ζ**_*t*_ from the individual sites to the *G* triangulation nodes (*j* = 1,….,*G*).

In addition to SST, PP, chlorophyll, geostrophic current velocities and distance from the nearest canyon, the model included year and month of sampling as covariates. Potential problems of multicollinearity among covariates were inspected through the variance inflation factor (VIF) [39]. Noninformative priors were used in the Bayesian computation. We used 170 randomly selected sites (70%) for calibration and the remaining 73 sites (30%) for validation of the model. Model performance was assessed through residual analysis and calculation of the actual coverage probability of a prediction interval with nominal coverage probability of 95%. Additional residual metrics included the root mean square error (RMSE) and the correlation between predictions and observations form the validation sites.

We explored variants of the model that considered the presence/absence rather than the number of outbreaks in each month (with binomial errors and logit link function), zero-inflated versions of the Poisson and binomial distributions and the zonal and meridional components of wind velocities instead of current velocities. These models yielded very similar results, so we will present results based on the Poisson distribution and geostrophic current velocities. This analysis was done using the INLA library in R [40].

### The environmental bootstrap

The estimated model was used iteratively with the environmental bootstrap method to determine the probability of observing a jellyfish outbreak under stochastic environmental fluctuations. The procedure started by smoothing the time series of environmental variables with a sliding window of 15 days (other choices of smoothing windows in the range of 9–31 days did not affect the results). The sliding window combined observations for the same period across years (for example, from 1 to 15 January for all years from 2004 to 2010) from which a mean and a standard deviation were obtained for the focal day of 8 January in this case. The mean was subtracted from the observed value and the difference was divided by the standard deviation to obtain a standardized residual for the focal day. The sliding window then moved to the next day and so on until the whole series of observations was smoothed (the leftmost and rightmost observations were repeated to fill the series at its boundaries).

The core of the environmental bootstrap method is the separation of the deterministic part of the signal from the stochastic component, the residuals, so that they can be randomized over time. Randomization is possible if residuals are statistically interchangeable, which implies that they are independent and identically distributed. Independence was assessed by estimating for each environmental variable the decorrelation time–i.e. the number of days at which the autocorrelation function reached zero. We used the number of days for the variable with the longest decorrelation time to define the length (in days) of the segments of standardized residuals to randomize. Standardized residuals had approximately mean zero and standard deviation of one within segments, so that they could be considered identically distributed for practical purposes. Another assumption is that the shape of the distribution of standardized residuals is not correlated with the smoothed means–i.e., the deterministic component of the signal. To assess this assumption we calculated an index of skewness as the ratio between the sample third central moment and the sample variance in each window of the series of standardized residuals, and inspected this index for any linear correlation with the smoothed means.

After randomization, the residuals were added back to the deterministic component of the signal. The first element in the randomized series was multiplied by the standard deviation calculated for the first point in the original time series and added to the expected value for that point, and so forth until a new hypothetical record of the time series was generated. This procedure was applied simultaneously to all the environmental variables and sampling sites, so that residuals were randomized in blocks to preserve the correlation structure among variables and the spatial correlation among sites.

One iteration of the procedure generates a hypothetical realization of environmental variables with the same predictable component of the original signal, but with environmental anomalies and extreme events reallocated randomly in time. A subset of the newly generated data matching the period of jellyfish sampling was then extracted from the randomized series and used as covariates to predict from the fitted model. Predictions were obtained by adding up the components of the linear predictor, the residuals and then back-transforming to the scale of the response variable.

We iterated the entire procedure 10000 times to calculate return times of jellyfish outbreaks using the generalized extreme value distribution (GEV) [1, 2]. The GEV distribution was fitted to the maximum value of jellyfish outbreaks observed over the 241 sites at each iteration, separately for each month. To compare the relative importance of deterministic and stochastic events, we randomized the deterministic component of the data in the same way as we did for the residuals. We then compared the standard deviations of jellyfish outbreaks obtained from the randomization of the stochastic and deterministic components of the environmental data. The rationale behind this analysis is that the randomization of a weak predictor will have little effect on the predicted values of the response, so that outcomes will be similar across bootstrapped replicates at any given site. In contrast, a strong predictor will generate more variability among bootstrapped replicates, with the predicted value of the response changing at any site in relation to the particular value of the predictor assigned to that site at each iteration. Standard deviations were obtained for each individual site (20 data points per site, one for each combination of year and month of sampling) across the 10000 boostraped replicates, and compared between the two randomization schemes with the Wilcoxon signed-rank test, matching data by site, year and month of sampling. Analyses were done in R [41].

## Results

**Fig 1:**
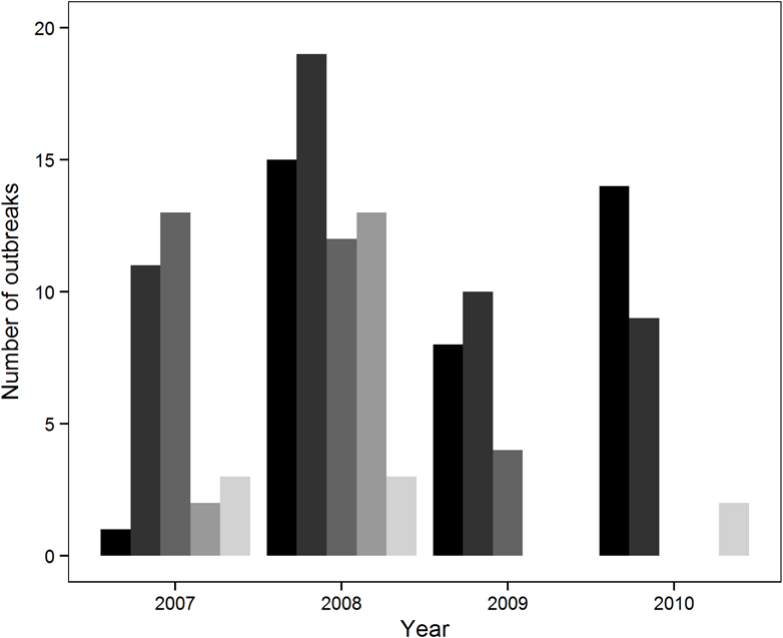
Timing of *Pelagia noctiluca* outbreaks along the Catalan coast. Bars from black to light grey correspond to sampling months from May to September in each year.

Stranding events with >1 *Pelagia noctiluca* jellyfish m^-2^ (outbreaks) were more frequent in May and June, particularly in 2008 (Fig 1). Outbreaks were negatively associated with SST and distance to the nearest canyon, and positively associated with the zonal and meridional components of geostrophic current velocities; all the posterior mean estimates of these covariates differed significantly from zero (Table 1). There was also a significant negative association between jellyfish outbreaks with year and month of sampling, reflecting the greater frequency of outbreaks in spring-early summer than in late summer, particularly in 2008 (Fig 1, Table 1). In contrast, the posterior coefficients for PP and chlorophyll did not differ significantly from zero (Table 1).

**Table 1:** Spatio-temporal Bayesian model of *Pelagia noctiluca* outbreaks along the Catalan coast.

Covariate	Mean	SD	Quantiles (95% credible interval)		
			0.025	0.5	0.975
Intercept	-0.4683	0.1716	-0.8048	-0.4684	-0.1315
Distance from nearest canyon	-0.0058	0.0028	-0.0113	-0.0058	-0.0003
Sea surface temperature	-0.0216	0.0075	-0.0364	-0.0216	-0.0069
Primary production	-0.0001	0.0002	-0.0005	-0.0001	0.0002
Chlorophyll *a*	-0.2680	0.2589	-0.7762	-0.2680	0.2399
Current zonal	0.0158	0.0062	0.0036	0.0158	0.0280
Current meridional	0.0188	0.0067	0.0057	0.0188	0.0319
Month	-0.0907	0.0224	-0.1343	-0.0908	-0.0464
Year	-0.0002	0.0001	-0.0004	-0.0002	-0.0001
$\sigma_{t}^{2}$	0.0670	0.0473	0.0832	0.0558	0.1759
$\sigma_{w}^{2}$	0.4424	0.1268	0.2576	0.4193	0. 7519
*ρ*	0.1219	0.0385	0.0642	0.1156	0.2146
*a*	0.5492	0.3169	-0.2109	0.6167	0.9549

The estimated model had a RMSE of 0.283 and the correlation between predicted and observed validation measures was 0.652. Actual coverage probability was 0.997, indicating that the model slightly overestimated the uncertainty of predictions. The variance inflation factor (VIF) was 2.4 for PP, indicating possible problems of multicollinearity. Removing PP from the analysis did not alter the effects of the other covariates, whose VIF values were lower than 1.5.

**Fig 2:**
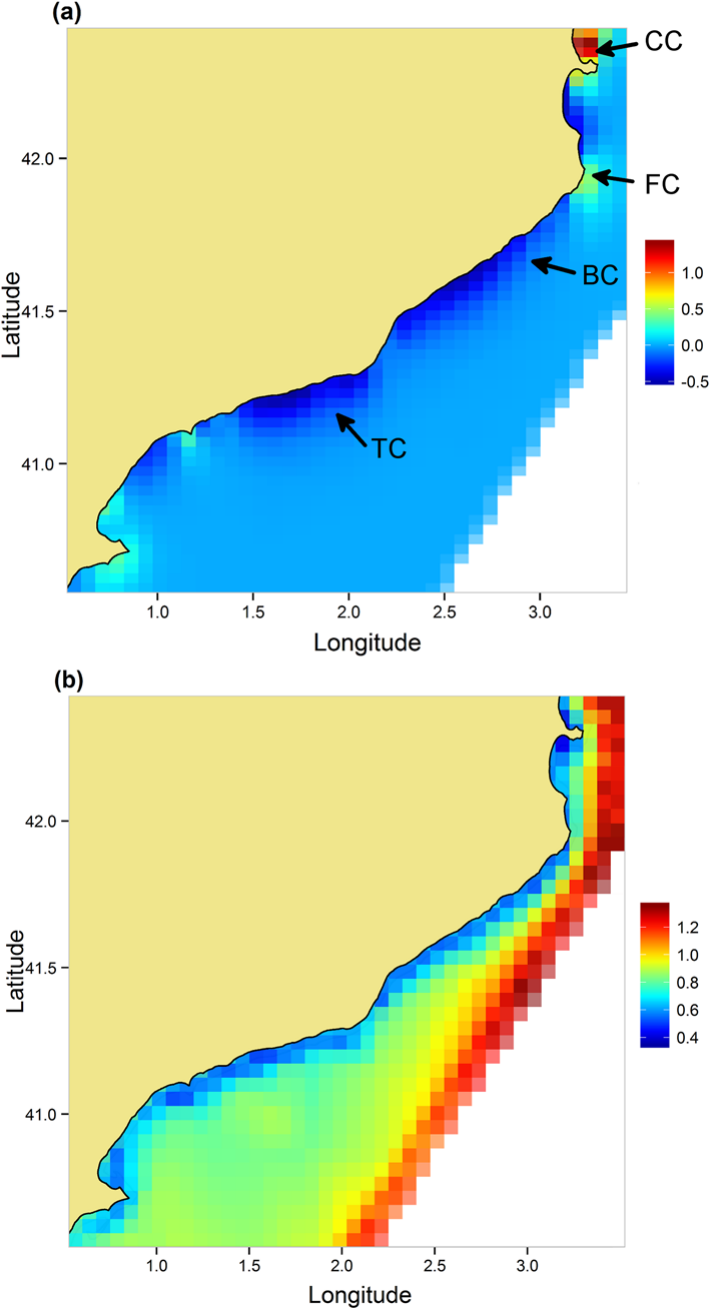
Gaussian Markov Random Field (GMRF) of *Pelagia noctiluca* outbreaks. Data are mean (a) and standard deviation (b) of the GMRF on the logarithmic scale. The GMRF extends seaward to cover the region defined by the Delaunay triangulation (S2 Fig). Arrows indicate the approximate location of canyons near the coast; from north to south: Cape De Creus (CC), Fondera (FC), Blanes (BC) and Tarragona (TC) canyon.

A map of the GMRF of *P*. *noctiluca* outbreaks indicated that the most affected locations were in the northern and, to a lesser extent, in the southern parts of the study region (Fig 2). Spatial correlation was low, but significant (*ρ* = 0.12), whereas first-order temporal autocorrelation was not significant (*a* = 0.55 with the 95% credible interval embracing zero (Table 1).

**Fig 3:**
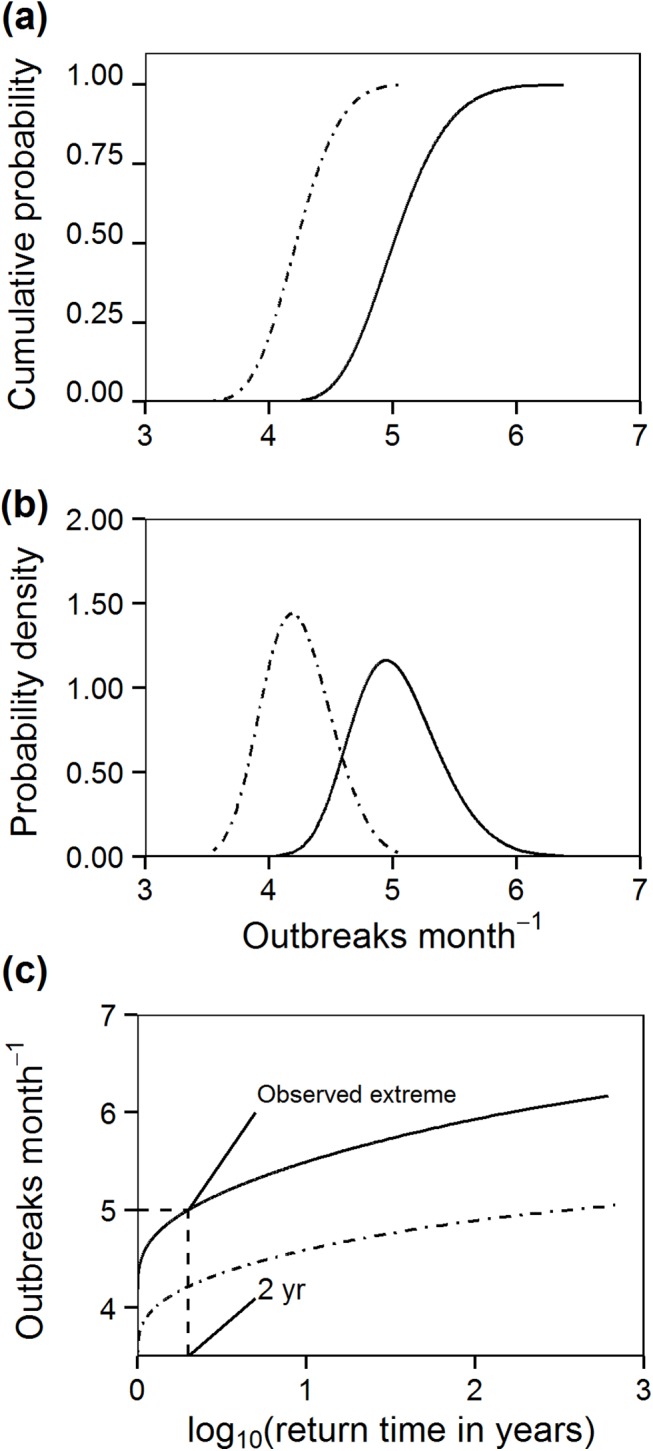
Extreme *Pelagia noctiluca* outbreaks. Empirical cumulative (a) and probability density (b) distributions and return time plot (c) for extreme *Pelagia noctiluca* outbreak events obtained from the environmental bootstrap analysis. Calculations are done for the month of May (dash-dot line) and June (continuous line) of a random year.

The environmental bootstrap method enabled us to calculate the cumulative probability function and the probability density curves of annual maxima (over the 241 sites) of *P*. *noctiluca* outbreaks. Results are illustrated for May and June when outbreaks were more frequent (Fig 3A and 3B). Outbreaks were more likely in June than May, but the difference was less than one event. This analysis also suggested a return time of two years for the maximum number of outbreaks at a given site in the empirical record (5 outbreaks observed in a month) (Fig 3C). The 100- and 1000-year return times were similar to the observed maximum number of outbreaks (Fig 3C).

**Fig 4:**
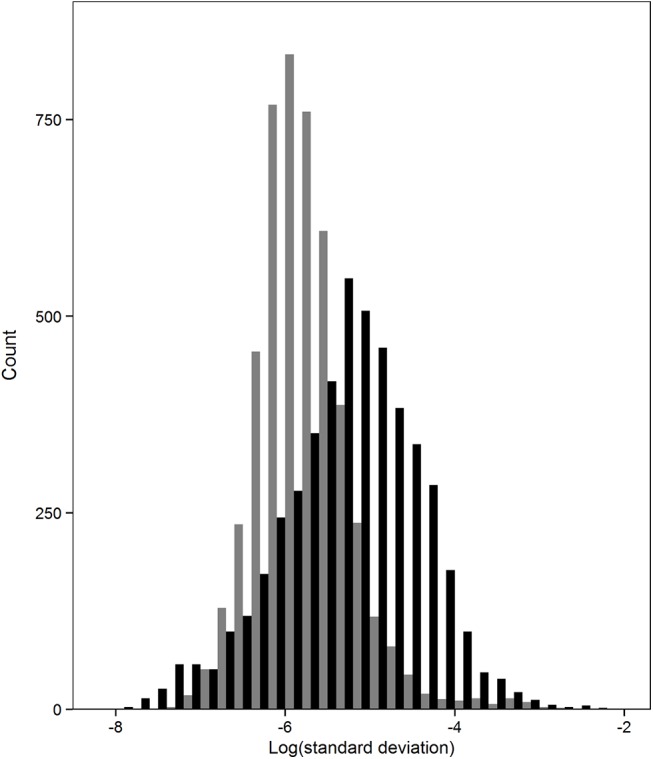
Deterministic vs. stochastic forcing. Frequency distributions of site standard deviations of *Pelagia noctiluca* outbreaks over 10000 bootstrapped replicates for the stochastic (grey bars) and deterministic (black bars) components of the environmental data.

Randomization of standardized residuals, as implemented in the environmental bootstrap method, resulted in lower standard deviations among bootstrap replicates than observed in the randomization of the deterministic component of the environmental predictors (Fig 4). The Wilcoxon signed-rank test of the null hypothesis that the data came from the same population was rejected (W = 1701, *P*<0.0001).

## Discussion

Jellyfish outbreaks may be a deterministic response to escalating levels of environmental degradation [16, 23]. A rising global trend in SST is a case in point. Several studies have reported the coincidence of greater abundances of jellyfish with warm conditions and experiments have shown how increasing temperatures may enhance asexual production in several gelatinous species [20, 32]. This evidence has raised concern that jellyfish outbreaks may become more frequent with global warming; nevertheless, temperature displays interannual variation within the long-term upward trend. Global climate change also includes many factors in addition to warming, such as increased frequency and intensity of extreme precipitation events, runoff and storms. In addition to climate change, other potentially important drivers of jellyfish outbreaks, such as fertilizers and organic pollution, habitat modification and overfishing are increasing globally in coastal regions (reviewed in 16). Although all these potential causes of jellyfish outbreaks have been acknowledged [24], a comprehensive understanding of the influence of individual drivers and their potential synergistic or antagonistic interactions has remained elusive. In principle, multiple causalities should make compounded effects and chance events more likely.

Our results indicated that deterministic processes were more important than the stochastic component of environmental variation. Thus, *P*. *noctiluca* outbreaks were unlikely to result from the chance coincidence of environmental events leading to favourable conditions for population renewal. SST and geostrophic currents were significant time-varying predictors, but their signal was not strong enough to avert deterministic effects. The negative relationship we found between *P*. *noctiluca* outbreaks and SST reflected seasonal effects likely linked to upwelling, with outbreaks being more frequent in cool waters during spring and early summer than in the warmer months. By contrast, the positive relationships between abundances of most jellyfish species and SST in the literature reflect interannual variability or regional effects [16, 32]. Our results were constrained by the sampling schedule, which excluded fall and winter months. Nevertheless, the seasonal fluctuations documented here were consistent with evidence that *P*. *noctiluca* numbers decline locally during the summer [8, 34], although this is not a general pattern throughout the Mediterranean [42]. Thus, our results apply to the dynamics of stranding during the summer season along the Catalan coast, which might be different in other periods of the year or in other regions.

We found a positive association between the zonal and meridional components of geostrophic currents and jellyfish outbreaks along the Catalan Coast. These results were consistent with the along-shore direction of the predominant flow in the study area, which is determined by the position of the Northern Current in the Ligurian Sea [43]. The continuous advection of cold waters from the Gulf of Lyons could also explain the negative relation between *P*. *noctiluca* outbreaks and SST [44]. The Gulf of Lyons is a very productive system and alongshore transport of *P*. *noctiluca* from this area might contribute to the number of outbreaks observed along the Catalan coast. Unfortunately, data on *P*. *noctiluca* in the Gulf of Lyons were not available to us, so this hypothesis awaits further testing.

Although the role of currents as drivers of concentration of gelatinous plankton has been widely recognized [45, 46], the precise mechanisms underlying cross-shore transport are still unclear [47–49]. The study region has a permanent shelf-front slope that separates saline open-sea waters from shelf waters with lower salinity. Previous studies have shown that *P*. *noctiluca* concentrates at the front, creating opportunities for cross-shore transport during relaxation events [48]. Our results were consistent with this view, emphasizing the importance of surface currents in driving jellyfish onshore.

Another study showed how canyons may deflect the predominant along-shore current in the onshore direction [49]. A recent hypothesis posits that canyons promote *P*. *noctiluca* aggregation and vertical migration so that outbreaks should be more frequent in coastal areas near these physiographic features of the sea floor [34]. Consistent with this hypothesis, we found fewer jellyfish outbreaks with increasing distance from canyons. Canyons may act as conveyor belts facilitating the transport of *P*. *noctiluca* from deep to shallow waters during upwelling and may provide corridors for deep-water delivery during cold-water cascading events. This hydrodynamic forcing may also strengthen the association between *P*. *noctiluca* and zooplankton food, whose availability may vary in space and time [50, 51]. Therefore, canyons may provide highly energetic habitats offering potentially important metabolic advantages to *P*. *noctiluca* [52].

However, jellyfish outbreaks only occurred in the proximity of the two northernmost canyons, whereas those located in the central part of the study area were unaffected (Fig 2A). In addition, outbreaks also occurred in the southern part of the study area, where there are no canyons. This patchiness underscores the influence of local oceanographic features and hydrological patterns that likely interact with topography or coastal heterogeneity in driving the spatial distribution of jellyfish outbreaks [53]. Taken together, these results suggest that the two northernmost canyons contributed disproportionately to *P*. *noctiluca* outbreaks and the jellyfish are subsequently distributed further south by currents.

We found no significant association between *P*. *noctiluca* outbreaks and chlorophyll. This was consistent with the results of other studies and it is not surprising, since chlorophyll concentration reflects standing biomass after removal processes such as grazing have been accounted for [32, 54]. As such, chlorophyll is not necessarily a good indicator of food availability (zooplankton) for gelatinous predators. PP, in contrast, reflects the rate of carbon fixation through photosynthesis and may provide a better indicator of food availability than chlorophyll. However, Lucas *et al*. [32] found that PP was not a particularly important predictor of the biomass of gelatinous zooplankton in the global ocean. In agreement with these findings, our analysis also indicated no significant relationship between PP and *P*. *noctiluca* outbreaks along the Catalan coast.

The environmental bootstrap method was developed to investigate the effects of stochastic extreme environmental fluctuations on ecological and biological response variables [7]. Denny *et al*. [7] used this procedure to assess the effect of extreme wave forces on mussel dislodgment and the influence of heat stress on limpet mortality on rocky intertidal shores. These analyses used mechanistic response functions to translate the distribution of maximum values of physical variables into meaningful biological responses. Instead of using a mechanistic response function, which was unavailable for *P*. *noctiluca* outbreaks, we used a statistical model to determine the likelihood that an extreme outbreak will occur by chance alone in a random year. This is a potentially important extension of the environmental bootstrap method, which can make this approach applicable to a wide range of ecological problems, without necessarily relying on the availability of mechanistic response functions or physiological models. Our results indicated that outbreaks were more likely in May and June than in any other summer month of a random year, as observed in the empirical data. Extreme value analysis showed that the largest number of observed outbreaks at a site (five events month^-1^) was a frequent event that can be expected to occur every two years. Furthermore, return time values indicated that the numbers of extreme outbreaks likely to be encountered in a century or millennium were only slightly larger than the number expected in a decade. Thus, the frequency of *P*. *noctiluca* outbreaks should not increase in the study region as a consequence of random environmental fluctuations.

Jellyfish populations often show a cyclic pattern of temporal variation with periods differing among species and locations, but that may involve decadal oscillations potentially related to climate [16, 22, 26, 27]. The data available to us did not allow evaluation of trends or periodicities; however, we observed *P*. *noctiluca* outbreaks throughout the study period with peaks varying between May and June depending on the year of sampling. These short term, often irregular events are less emphasized in the literature than the more regular temporal patterns, but interannual variation is often dramatic in jellyfish populations [16, 18].

Deterministic environmental factors such as shelf topography, geomorphology and possibly other local hydrological processes appeared more important than stochastic environmental fluctuations in driving *P*. *noctiluca* outbreaks. Our results support the hypothesis that canyons can funnel *P*. *noctiluca* blooms towards shore during upwelling [34]. This is consistent with the occurrence of jelly-carbon depositions in canyons [55]. Notwithstanding the importance of deterministic environmental factors, species undergoing outbreaks must possess appropriate life-history traits to enable rapid population growth and quick response to environmental change [56]. Thus, demographic and population-level processes should be regarded as the proximate causes of outbreaks. Most jellyfish species reproduce asexually, which allows for exceptionally high growth rates and the presence of a diapause stage increases tolerance to stressful environmental conditions (e.g. anoxia) [16–18, 23, 57]. A better understanding of how environmental factors affect these population and demographic processes can greatly improve our ability to anticipate jellyfish outbreaks in the future.

## Supporting Information

S1 Data Data on *Pelagia noctiluca* outbreaks. (CSV)

S1 Fig Comparison of number of outbreaks estimated from stranded data and from boat observations. (PDF)

S2 Fig Constrained refined Delaunay triangulation of the study region. (PDF)

S1 References References included in S1 Table. (PDF)

S1 Table Source and extent of environmental data. (PDF)
