# Supplementary material for: Deterministic Factors Overwhelm Stochastic Environmental Fluctuations as Drivers of Jellyfish Outbreaks
Source: PLoS One. 2015 Oct 20;10(10):e0141060. doi: 10.1371/journal.pone.0141060 (PMC4617864; doi:10.1371/journal.pone.0141060)
Supplement: S1 Fig — (PDF) [file pone.0141060.s002.pdf]

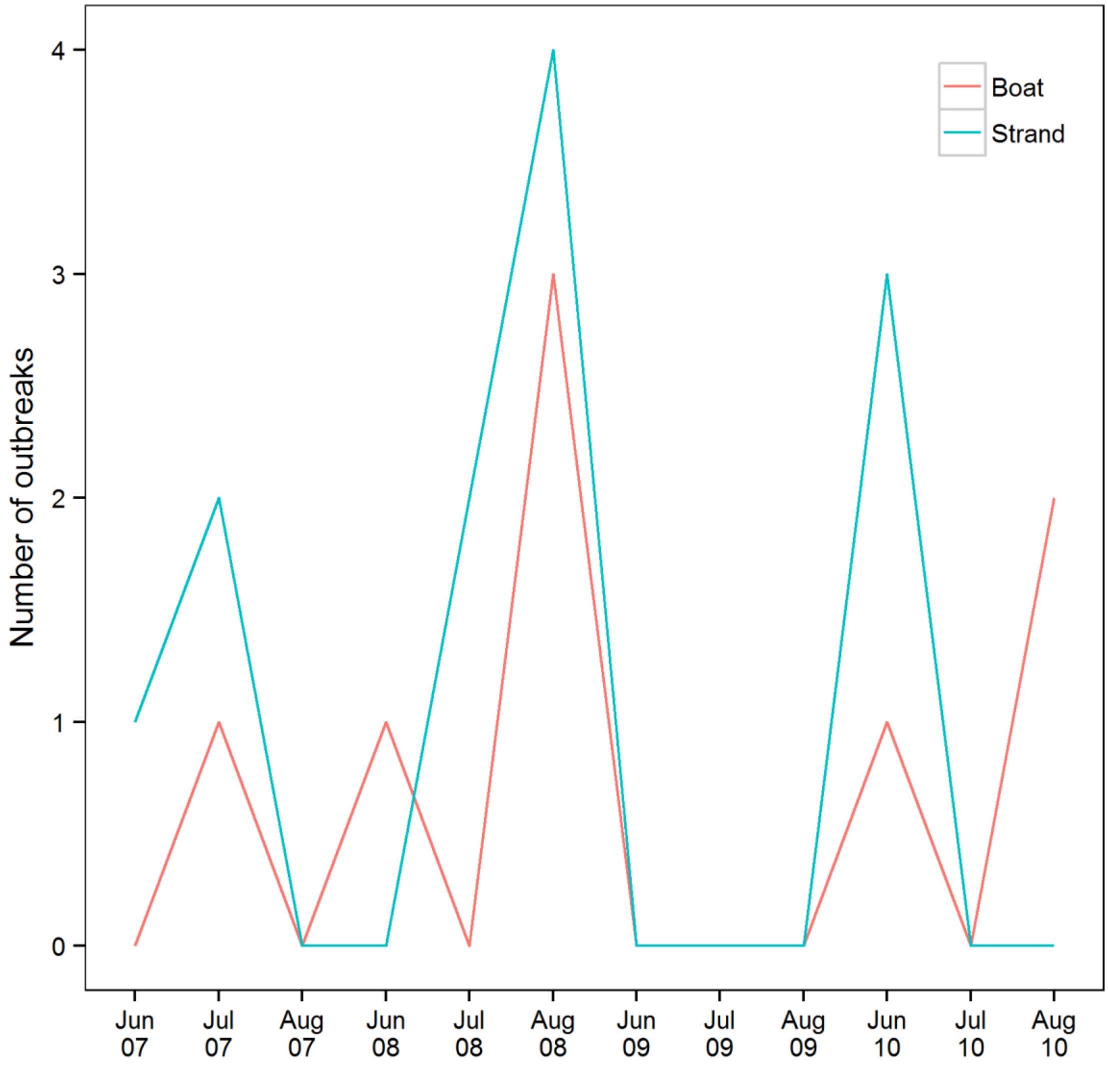

Fig. S1. Comparison of number of outbreaks estimated from stranded data and from boat observations at the 10 sites most affected by *Pelagia noctiluca* blooms. Stranded data are temporally independent in the sense that beaches were cleaned daily so that only new arrivals have been counted each day. Boat observations apparently underestimate the incidence of outbreaks compared to measurements based on stranded data. A formal cross-correlation analysis was not done because sampling was not performed at regular intervals.
