# Supplementary material for: Deterministic Factors Overwhelm Stochastic Environmental Fluctuations as Drivers of Jellyfish Outbreaks
Source: PLoS One. 2015 Oct 20;10(10):e0141060. doi: 10.1371/journal.pone.0141060 (PMC4617864; doi:10.1371/journal.pone.0141060)
Supplement: S2 Fig — (PDF) [file pone.0141060.s003.pdf]

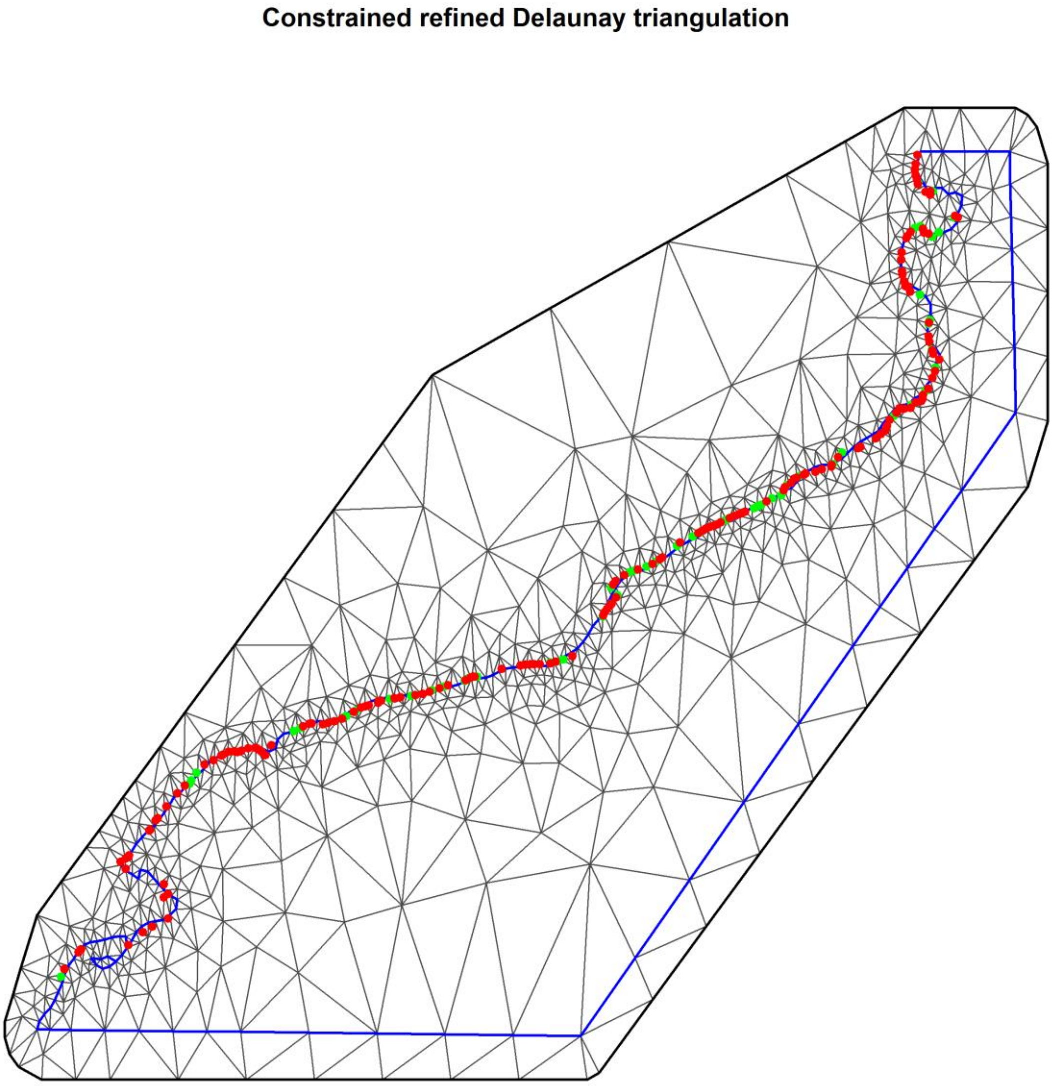

Fig. S2. Constrained refined Delaunay triangulation of the study region (Catalan coast, Spain). Red and green dots are *Pelagia noctiluca* calibration and validation sampling sites, respectively. The blue line delimits the border of the projection region.
