# Supplementary material for: Deterministic Factors Overwhelm Stochastic Environmental Fluctuations as Drivers of Jellyfish Outbreaks
Source: PLoS One. 2015 Oct 20;10(10):e0141060. doi: 10.1371/journal.pone.0141060 (PMC4617864; doi:10.1371/journal.pone.0141060)
Supplement: S1 References — (PDF) [file pone.0141060.s004.pdf]

Supporting References

Chin TM, Vazquez J, Armstrong E, Mariano A (2010) Algorithm theoretic basis document: multi-scale, motion-compensated analysis of sea surface temperature Version 1.3. Available online: *ftp://mariana.jpl.nasa.gov/mur_sst/tmchin/docs/ATBD/atbd_1.3.pdf*

Maritorena S, Hembise Fanton d'Andon O, Mangin A, Siegel DA (2010) Merged satellite ocean color data products using a bio-optical model: characteristics, benefits and issues. *Remote Sensing of Environment*, **114**: 1791-1804
