# Supplementary material for: Deterministic Factors Overwhelm Stochastic Environmental Fluctuations as Drivers of Jellyfish Outbreaks
Source: PLoS One. 2015 Oct 20;10(10):e0141060. doi: 10.1371/journal.pone.0141060 (PMC4617864; doi:10.1371/journal.pone.0141060)
Supplement: S1 Table — (PDF) [file pone.0141060.s005.pdf]

Table S1. Source and extent of environmental data

Variable	Source	Temporal extent	Spatial resolution
**Sea Surface Temperature**	GHRSST Level 4 MUR (Chin et al. 2010) http://podaac.jpl.nasa.gov/dataset/JPL-L4UHfnd-GLOB-MUR	01/05/2004 30/09/2010	1 km
**Geostrophic currents**	AVISO product http://www.aviso.oceanobs.com/index.php?id=1275 *(The altimeter products were produced by Ssalto/Duacs and distributed by Aviso, with support from Cnes http://www.aviso.altimetry.fr/duacs/)*	01/05/2004 30/09/2010	1/8°x1/8° (14 km)
**Primary production**	Standard VGPM data http://orca.science.oregonstate.edu/2160.by.4320.monthly.hdf.vgpm.m.chl.m.sst4.php	01/05/2004 30/09/2010	9 km
**Chlorophyll *a* concentration**	Ocean Color MEaSUREs project (Maritorena et al. 2010) http://wiki.icess.ucsb.edu/measures/index.php/GSM	01/05/2004 30/09/2010	9 km
